# Generation of propulsive force via vertical undulations in snakes

**DOI:** 10.1242/jeb.239020

**Published:** 2021-07-06

**Authors:** Derek J. Jurestovsky, Logan R. Usher, Henry C. Astley

**Affiliations:** Department of Biology, University of Akron, 302 E. Buchtel Avenue, Akron, OH 44325, USA

**Keywords:** Propulsive impulse, Limbless locomotion, Robotic model

## Abstract

Lateral undulation is the most widespread mode of terrestrial vertebrate limbless locomotion, in which posteriorly propagating horizontal waves press against environmental asperities (e.g. grass, rocks) and generate propulsive reaction forces. We hypothesized that snakes can generate propulsion using a similar mechanism of posteriorly propagating vertical waves pressing against suitably oriented environmental asperities. Using an array of horizontally oriented cylinders, one of which was equipped with force sensors, and a motion capture system, we found snakes generated substantial propulsive force and propulsive impulse with minimal contribution from lateral undulation. Additional tests showed that snakes could propel themselves via vertical undulations from a single suitable contact point, and this mechanism was replicated in a robotic model. Vertical undulations can provide snakes with a valuable locomotor tool for taking advantage of vertical asperities in a variety of habitats, potentially in combination with lateral undulation, to fully exploit the 3D structure of the habitat.

## Abstract (precis)

**Summary:** Snakes are capable of generating propulsion via vertical undulations, which allows them to exploit their environment in 3D and allows more effective use of previously overlooked surfaces in cluttered habitats.

## INTRODUCTION

All animals achieve locomotion by applying force to the environment, thereby generating reaction forces which propel the animal (Dickinson et al., 2000). Limbed vertebrates typically have discrete propulsive contact points (feet), which must simultaneously generate forces to support body weight, provide propulsive force and maintain stability. However, in terrestrial limbless vertebrates, any body segment can be propulsive, while stability and support needs are minimal in most environments and frictional forces overwhelm inertial effects (Gray, 1946; Hu et al., 2009). Terrestrial limbless vertebrates propel themselves using a wide range of locomotor modes, depending upon the type of environment they encounter; however, lateral undulation is the most common across and within taxa (Gans, 1962). Lateral undulation uses posteriorly propagating horizontal waves of bending that contact and push against asperities in the environment (e.g. grass, rocks, sticks), generating reaction forces that propel the animal forward (Gans, 1962; Gray and Lissmann, 1950; Jayne, 1986).

**Fig. 1.**
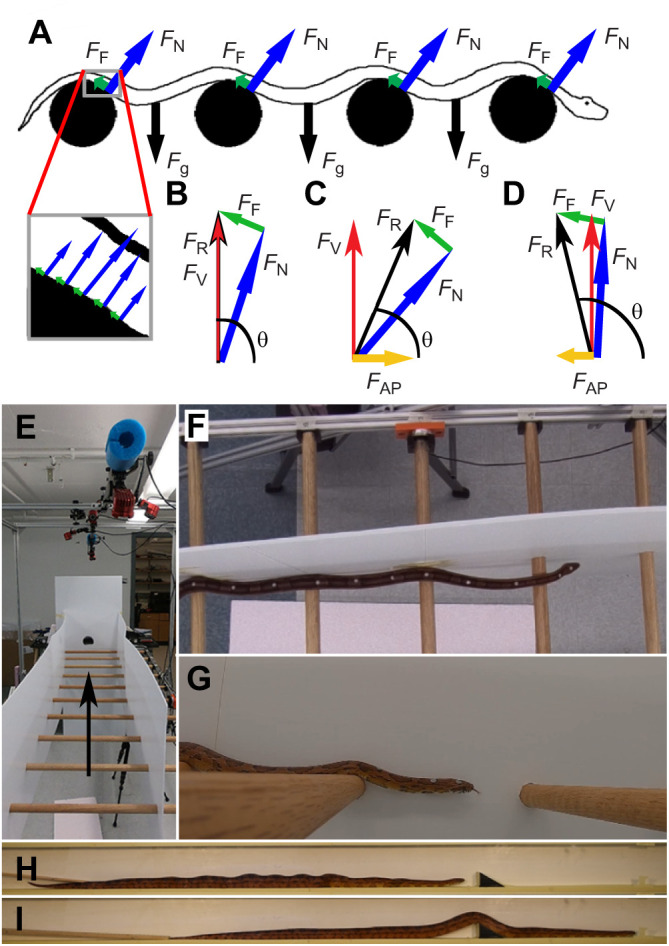
**The experimental setup and still images from trials.** (A) A lateral-view diagram of a snake using vertical undulations across multiple dowels (black circles), showing idealized forces. Inset shows the force distribution across the contact surface, which is summed into an overall reaction force in the main image. (B) Diagram of reaction forces for a snake progressing at constant velocity. Because there is no net acceleration, there is also no net force. (C) Diagram of reaction forces if the snake is accelerating, generating a net forward force. (D) Diagram of reaction forces if the snake is decelerating, generating a net braking force. *F*_AP_, anteroposterior force; *F*_F_, frictional force; *F*_g_, force due to gravity; *F*_N_, normal force; *F*_R_, resultant force; *F*_V_, vertical force; θ, angle of *F*_R_. (E) Experimental setup showing cameras overhead and the horizontal ladder, with an arrow indicating the direction of movement of the snake. (F) Dorsal view of a corn snake using vertical undulations. The body is close to but not in contact with the side wall. (G) Lateral view of a corn snake using vertical undulations (Movie 1). (H,I) Side views of a corn snake moving through a tunnel with a single potential contact for vertical undulations (Movie 2). The snake initially performs concertina locomotion (H), indicated by the tight body waves, but switches to vertical undulations (I) once it has sufficient contact with the wedge.

Snakes are also capable of generating propagating vertical waves, observed during lateral undulation and sidewinding to reduce friction on certain body segments (Hu et al., 2009; Marvi et al., 2014) and during gliding for stabilization (Yeaton et al., 2020). However, the ability of snakes to generate propulsive reaction forces from vertical waves in terrestrial environments has never been tested. We hypothesized that snakes can use vertical waves to generate propulsive forces via a similar mechanism to lateral undulation when in contact with vertical asperities in the substrate at suitable angles (Fig. 1A–D). To test our hypothesis, we measured substrate reaction forces and kinematics as snakes traversed an experimental setup designed to elicit this behavior while impeding other modes of locomotion and confounding factors. Furthermore, our hypothesis predicts that snakes should only be able to generate net propulsive forces from surfaces with a vertical slope beyond the angle of frictional slipping (Fig. 1B–D); thus, we tested the snake in an experimental setup with only a single potential propulsive surface oriented at an angle predicted to be either sufficient or insufficient for propulsion via vertical undulation. Finally, we attempted to replicate propulsion via vertical undulation in a robotic model to show that observed propulsion in the snake is not attributable to unobserved mechanisms, and that the proposed mechanism is mechanically sound even in the absence of snake musculoskeletal anatomy and neural control.

## MATERIALS AND METHODS

Four adult wild-caught corn snakes, *Pantherophis guttatus* (Linnaeus 1766), were obtained from a commercial provider [mean±s.d. snout–vent length (SVL) 102.4±9.3 cm, range 92.6–114.3 cm; mass 463±62.6 g, range 340–550 g). This species was chosen because they are locomotor generalists and thus likely to elicit the desired behavior. All experiments were approved by University of Akron IACUC. Locomotion trials were conducted after warming the snakes to 29–32°C, the field active temperature of a congeneric (Brattstrom, 1965).

We constructed a 248 cm long trackway consisting of a frame of 80/20 longitudinal supports with 11 horizontal oak dowels (91 cm long, 2.5 cm diameter) placed perpendicular to the longitudinal supports and spaced at 20 cm intervals, much like the rungs of a ladder laid horizontally (Fig. 1E). Walls were placed 45 cm apart on either side of the dowels (walls extended 36.5 cm above the dowels and 22.0 cm below) and the trackway was raised 88 cm above the ground to dissuade the snakes from leaving the trackway. Oak dowels were sanded and treated with a polyurethane sealant. Snakes were induced to move along the length of the trackway and thus perpendicular to the dowels (Fig. 1F,G). Trials were performed in sets of three per 24 h and individuals were allowed a minimum of 5 min rest between trials to prevent fatigue. A dark enclosure was placed at the end of the trackway to encourage movement in the desired direction and to allow for a location of rest between trials. Light tapping, rubbing with fingers or touching with a snake hook was used on the tail to encourage movement, though we did not attempt to induce maximal speed from the animals. Snakes were not tested for 24 h after feeding occurred. To provide an experimental control and clear contrast between the forces produced in active versus passive systems, and to show that our data are not an artefact of our measurement system, we dragged a braided nylon rope (229 g, 144 cm long, 1.7 cm diameter) across the dowel array, as this should produce only braking force and braking impulse. The coefficient of friction was measured using a standard tilting plane method, in which snakes were conscious and alert. The snakes were oriented with most body segments parallel to the slope with anterior downwards (the presence of body segments at other angles would slightly over-estimate the coefficient of friction as a result of scale anisotropy) on a plane of oak prepared identically to the dowels and tilted until they began to slide (Astley and Jayne, 2007; Gray and Lissmann, 1950; Sharpe et al., 2015); the average coefficient of friction was 0.17±0.02 (range 0.14–0.19) for the snakes (*n*=4) and 0.28±0.03 (range 0.23–0.32) for the rope based on 3 trials per individual/object. While there were some trials in which only braking force was recorded, to streamline analysis, only trials with propulsive force were analyzed (see Results and Discussion).

Two six-axis force/torque sensors (Nano 43, ATI Industrial Automation, Apex, NC, USA) were connected on either end of a single dowel mid-way along the trackway (dowel 6 of 11). Outputs of the force sensors were collected using 12 channels (six per sensor) on a NIDAQ N1-USB-6218 (16 bits, National Instruments, Austin, TX, USA) and recorded using the software IGOR Pro (WaveMetrics, Tigard, OR, USA) at 1 kHz. This force-sensing dowel was calibrated using hanging masses and pulleys at different angles and locations along the dowel to apply known anterior/posterior, lateral and vertical forces, which were used to create a calibration matrix using the MATLAB function linsolve (MathWorks, Natick, MA, USA). Force data were splined to smooth the data in IGOR Pro, and analyzed using a custom-written script in MATLAB. Data were normalized to body weight to facilitate comparisons between individuals (Fig. S1). During rope trials, forces induced by inertial motion of the end of the rope dropping from an adjacent dowel would confound analysis; thus, we only included the smooth rise and steady state of the forces during these trials (Fig. S2). The impulse (the time integral of force, in BW s) is the total change in momentum of the system, and was used to determine whether the overall interaction between the snake and the force-sensing dowel had a net propulsive or net braking effect, similar to studies of limbed animals (Budsberg et al., 1987; Hodson et al., 2001).

Kinematics were recorded at 120 images s^−1^ using six motion capture cameras (Flex 13, NaturalPoint, Inc., Corvallis, OR, USA) placed 1 m above the dowels at varying angles (Fig. 1E). Small markers of infra-red 7610 reflective tape (3M, St Paul, MN, USA) were placed at regular intervals (∼10 cm) along the dorsal side of each snake. Camera synchronization, recording, calibration, point tracking and position calculation were all accomplished using Motive Optitrack software v.2.0.2 (NaturalPoint, Inc.), which then exported 3D marker coordinates. A HERO6 Black GoPro (GoPro Inc., San Mateo, CA, USA) camera was also used to record video from above for visual confirmation, but not analysis. To determine how straight the snake was when moving across the force-sensing dowel (and thereby rule out lateral undulation), we analyzed motion capture data (dorsal view, fore–aft and lateral components) using a custom-written script in MATLAB to perform a linear regression on the points within 20 cm of the force dowel throughout the trial. The captured region spanned three dowels (middle dowel with the force sensors) while the entire snake's body contacted between five and six dowels at any one time. Snakes occasionally used lateral bends prior to and after this region; however, trials were discarded if any lateral bends occurred on the force-sensing dowel or adjacent dowels. We quantified the maximum residual and the 95% confidence interval of the residuals as metrics of body straightness, and the angle of the body relative to the trackway (θ ϕ=0 deg is parallel and ϕ=90 deg is perpendicular). To quantify the vertical undulations along the captured region, we analyzed the motion capture data (lateral view, vertical and fore–aft components) by splining along the captured region, normalized the splines by height at the force-sensing dowel, and ran both an ANOVA and Tukey's (5% probability) *post hoc* statistical tests using custom-written scripts in MATLAB. Overall velocity was calculated from Motive Optitrack data in the horizontal plane, fore–aft and lateral components.

The snake exerts a net normal and frictional force on the dowel, with the normal force being perpendicular to the substrate and the frictional force being tangent and equal to the magnitude of the normal force multiplied by the coefficient of friction (µ) (Fig. 1B–D). The vector sum of the normal force and frictional force is the net substrate reaction force, the angle of which determines whether there is net propulsive or braking force (Fig. 1B–D). The force sensors in our study provide us the antero-posterior (*F*_AP_) and the vertical (*F*_V_) components of this reaction force (Fig. 1A–D). Based on these relationships (see Appendix for derivations of equations), one can calculate the magnitude of the normal force (*F*_N_):(1)
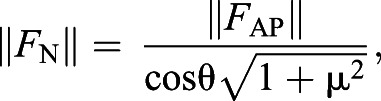
where θ is the angle of the resultant force (*F*_R_):(2)
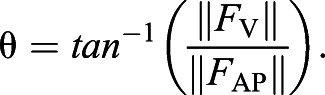
From these equations (and those easily derived from them), the magnitude and orientation of any of the vectors can be derived (Fig. 1B–D); however, we report the antero-posterior (*F*_AP_) and the vertical (*F*_V_) forces (particularly *F*_AP_), as these components directly test our hypothesis.

To test whether single vertical asperities of the appropriate orientation could be used to generate propulsion in a terrestrial setting (despite drag on many body segments), we constructed a trackway tunnel made of 1.27 cm thick expanded PVC boards, a common construction material consisting of a foamed PVC interior with a smooth surface finish. This trackway was 5 cm wide and 180 cm long with a sloped wedge three-quarters of the way along the trackway (Fig. 1H,I). All horizontal surfaces were covered with masking tape, which had an average coefficient of friction with the snakes of µ_tape_=0.21±0.06. One lateral wall was clear acrylic, and video was recorded using a Nikon D3300 DSLR camera (Nikon, Tokyo, Japan). In one set of trials, the wedge had a slope of 30 deg, steeper than the predicted minimum necessary for propulsive force [tan^−1^(µ_tape_)=11.3 deg] and thus suitable for generating propulsive forces (Fig. 1C; Fig. S4), while in the second set of trials, the wedge had a slope of 8 deg, which is predicted to be insufficient for generating net propulsive force (Fig. 1D; Fig. S4). Each snake moved through the tunnel 3 times, separated by rest periods of at least 15 min.

To test whether pure vertical undulation is sufficient to traverse our experimental setup and rule out unobserved mechanisms, a 13-link snake robot composed of 12 servo-motors (Hitec HS-85BB, Hitec RCD USA, Inc., Poway, CA, USA) mounted in custom 3D-printed brackets was constructed (total length 73.5 cm, mass 398.6 g, coefficient of friction 0.47±0.03, range 0.45-0.53). The snake robot was controlled through a USB servo controller (Lynxmotion, SSC-32U, Robotshop, Mirabel, QC, Canada) using a custom-written Python script (Python Software Foundation, Wilmington, DE, USA; see Supplementary Materials and Methods 1) using a serpenoid wave (Hirose, 1993) with the equation:(3)
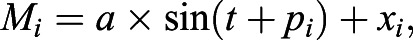
where *M_i_* is the angle of motor *i*, *a* is the maximum angle possible, *t* is time, *p_i_* is phase shift between successive motors, and *x_i_* is an offset to ensure all links are parallel when all motors are at an angle (*M*) of zero. The values used for these experiments were *a*=600 µs (for pulse-width modulation control) and *P*=1.5 radians, which produced two waves on the body and a suitably long, shallow wave to span two or more pegs in order to support itself (Movie 3). The robot had no sensors and body posture was under open-loop control.

## RESULTS AND DISCUSSION

**Fig. 2.**
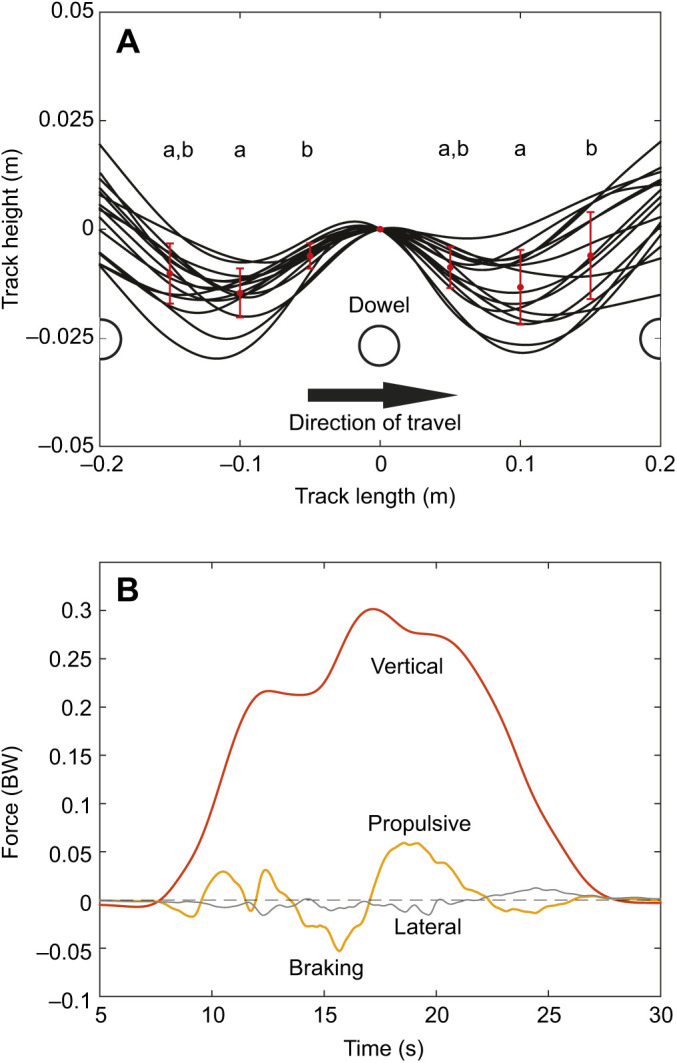
**Analysis of snake movement through the apparatus.** (A) Splines of dorsal marker paths in all trials (lateral view). Red dots and bars indicate the mean and s.d. of vertical displacement at 5 cm intervals (relative to the midpoint), showing clear vertical displacement prior to and after the force-sensing dowel (zero), represented by the white circle. Lowercase letters reflect significant differences based on Tukey's *post hoc* test and the arrow indicates the direction of movement. (B) Forces during a complete vertical undulation trial, from initial head contact (∼7 s) until the tail has lost contact with the force-sensing dowel (∼27 s). The corn snakes were spread over 4–6 pegs and the weight was unevenly distributed along its length. The orange line is the vertical force and the yellow line is the anterior/posterior force. The gray line is the lateral force and the dashed line marks zero force. Propulsive force is positive, while braking force is negative. BW, body weight.

Snakes were able to move across the setup using propulsive vertical undulations (mean±s.d velocity 0.04±0.03 SVL s^−1^, 4.1±2.6 cm s^−1^) despite minimal lateral undulation and no apparent use of other modes (Movie 1). The motion capture data revealed significant vertical displacement of the body across the force-sensing dowel (*F*_5,90_=4.37, *P*<0.0013) (Fig. 2A) with a nearly straight horizontal posture approximately parallel to the trackway (2.18±2.26 deg relative to the trackway), and that most points followed a nearly straight path (maximum lateral excursion 11 cm, 95% confidence interval 3.0 cm). Snakes applied highly variable forces to the instrumented dowel, ranging from pure braking to predominantly propulsive, without clear temporal patterns (Fig. 2B; Fig. S1). The maximum propulsive force on a single peg (0.08±0.04 body weight, BW) was larger than the maximum braking force on a single peg (−0.04±0.02 BW) in all trials analyzed (Table 1); the maximum frictional force to be overcome is 0.17 BW for the entire snake. The maximum lateral force in either direction was small (0.02±0.01 BW). For all but two trials, there was a net propulsive impulse (Table 1), and the average propulsive impulse was more than double the braking impulse (Table 1). The average lateral impulse was low (Table 1). The control trials in which a rope was dragged across the trackway generated high maximum braking force (−0.09±0.006 rope weight, RW), consistent with the higher coefficient of friction, but never generated propulsive force (Table 1). The average lateral force in either direction of the rope trials was low (0.01±0.006 RW). The rope had purely braking impulse (Table 1) and the average lateral impulse of the rope was low (Table 1).

**Table 1.**
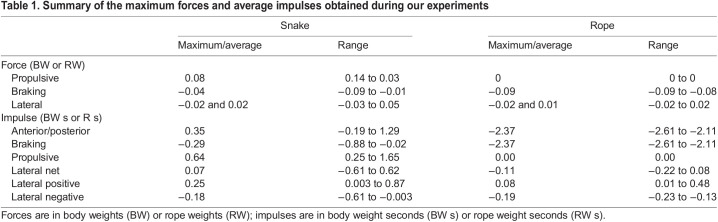
Summary of the maximum forces and average impulses obtained during our experiments

In the trials within the tunnel, the corn snakes always used concertina locomotion prior to encountering the sloped surface, but noticeably transitioned to vertical undulations shortly after encountering the 30 deg sloped wedge in all but two (10/12) of the trials (Fig. 1I; Movie 2). In contrast, the snakes encountering the 8 deg sloped wedge continued to perform concertina locomotion across the wedge, and never used vertical undulations (Movie 2).

The snake robot successfully moved across the setup using a vertical waveform in all 5 trials attempted (Movie 3). Because the snake robot consisted of rectangular body segments connected by revolute joints, once a given segment achieved sufficient contact angle to generate propulsive force (Fig. 1D), the robot would slide forward until the subsequent segment (with insufficient angle) collided with the dowel. This resulted in a discontinuous velocity, which, in turn, precluded effective force measurements.

### Conclusions

These results confirm our hypothesis that snakes can generate propulsive force via posteriorly propagating vertical waves down the body (Fig. 1A–D), albeit in a highly constrained, artificial system. During all trials, snakes had a relatively straight posture with minimal lateral bending across the region with the force-sensing dowel. This posture precludes the use of lateral undulation to generate the observed forces; inspection of video recordings showed no evidence of rectilinear movement. Similarly, while snakes may use rib motions or muscular connections to and within the skin to deform the ventral surface during this behavior, the robotic trials show that the proposed mechanism can function effectively even in a highly simplified system without these anatomical benefits.

Several lines of evidence suggest that snakes can generate considerable propulsive force per contact via vertical undulations. In several un-analyzed trials, no propulsion was captured by the force-sensing dowel but steady forward progression was nonetheless occurring without obvious lateral undulation (Fig. S3). As we were only able to measure forces at a single dowel, this suggests that snakes do not need to use every contact point to propel themselves using vertical undulations, and trials without measured propulsion were generating propulsion using other contact points. Consistent with this, the mean peak propulsive force across trials was 0.08 BW, almost half the force necessary to propel the snake (given a coefficient of friction of 0.17), with one trial showing a force of 0.14 BW, indicating that snakes were capable of generating sufficient force for propulsion from as few as two contact points. Similarly, during the tunnel trials, the entire snake was propelled via a single contact area on the 30 deg inclined wedge.

While snakes are unlikely to use purely vertical undulations to move through their environment, propulsive vertical undulations (as opposed to drag-reducing vertical motion in sinus lifting and sidewinding; Hu et al., 2009; Marvi et al., 2014) could be easily combined with lateral undulation. Snakes might use lateral undulation until they encounter a vertical asperity, then use vertical undulations against this object while simultaneously using lateral undulation at other points on the body, as opposed to simply dragging their body across these vertical obstructions. This mechanism has the potential to be particularly advantageous in arboreal locomotion, where a variety of structures provide useful contacts for vertical undulations. Similarly, rodent burrows are often spatially complex and vertical undulations could also be employed if suitable asperities are present, as in the tunnel trials, rather than using concertina locomotion as snakes typically do in narrow, flat tunnels.

Our experiments confirm that snakes can use vertical undulations to propel themselves, but whether this mechanism can be classified as a new mode of locomotion is uncertain. Jayne (2020) highlights at least 11 modes of locomotion under four specific headings (i.e. rectilinear, sidewinding, five types of lateral undulation and four types of concertina). Vertical motion has been previously documented in lateral undulation and sidewinding for reducing friction (Hu et al., 2009; Marvi et al., 2014) and during gliding for stability (Yeaton et al., 2020) but never previously for direct generation of propulsive force. However, while we demonstrate effective locomotion using only vertical undulations, our instrumented trackway is, by necessity, a highly constrained and artificial system, and snakes are unlikely to use purely vertical undulation in natural environments. Instead, vertical undulations may be combined with lateral undulation during terrestrial and arboreal locomotion when sufficient vertical asperities are present, or used in an intermittent, non-cyclic form, as in our tunnel trials. However, whether or not this mechanism is a true ‘mode’ of locomotion, the ability of snakes to use vertical undulations to generate propulsion dramatically expands our understanding of snake locomotor mechanics and their interactions with their habitats. By using vertical undulations, snakes demonstrate the ability to exploit the complexity of their habitat in three dimensions, generating propulsive forces from previously overlooked surfaces and allowing more effective use of cluttered habitats.

## Supplementary Material

Supplementary information

## APPENDIX

The derivation of Eqns 1 and 2 is based on terminology and forces from Fig. 1A–D.

Derivation of Eqn 1:

(A1)

(A2)

(A3)

(A4)

(A5)

(A6)

(A7)

(A8)

where *F*_AP_ is anteroposterior force, *F*_F_ is frictional force, *F*_N_ is normal force, *F*_R_ is resultant force and θ is the angle of *F*_R_.

Derivation of Eqn 2 is based on the triangle made with *F*_R_, *F*­_V_ and *F*_AP_ (Fig. 1):

(A9)

(A10)

where *F*_V_ is vertical force.
