# Supplementary material for: Generation of propulsive force via vertical undulations in snakes
Source: J Exp Biol. 2021 Jul 6;224(13):jeb239020. doi: 10.1242/jeb.239020 (PMC8278011; doi:10.1242/jeb.239020)
Supplement: Supplementary information [file jexbio-224-239020-s1.pdf]

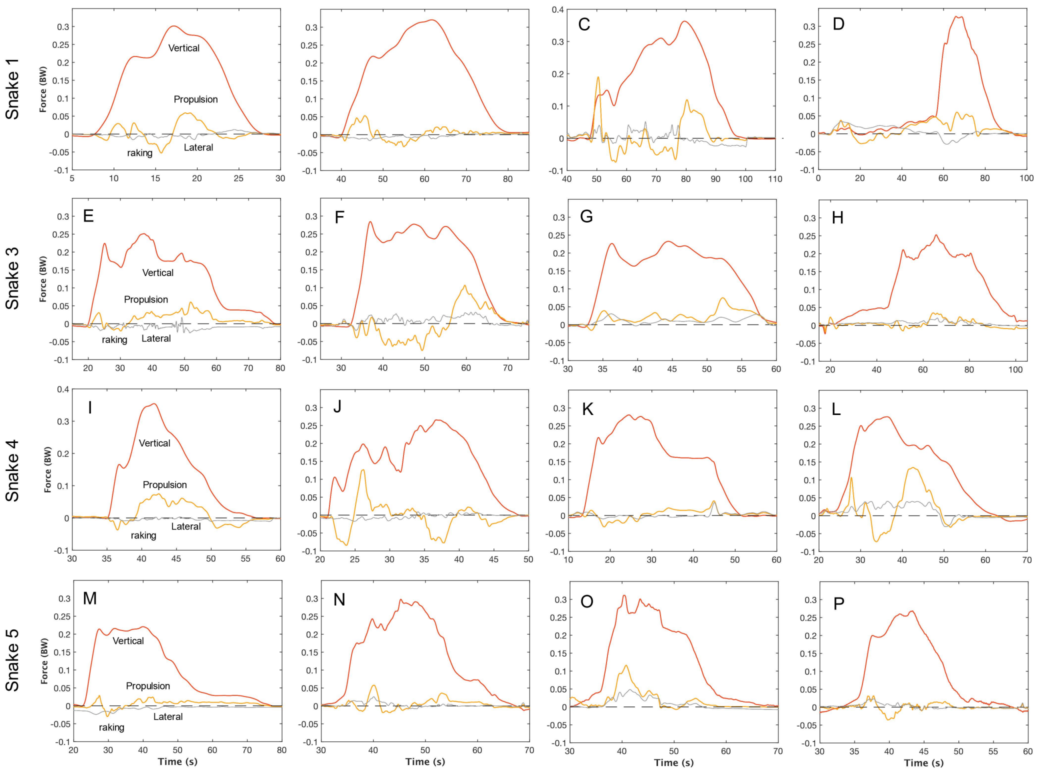

Fig. S1. All trials we obtained showing the forces (BW) as the snake passed along the force-sensing dowel. Trials involving snake 1 are represented by A-D, snake 3 E-H, snake 4 I-L, and snake 5 M-P. Snakes 2 and 6 were not included as they would not cooperate.

Journal of Experimental Biology • Supplementary information

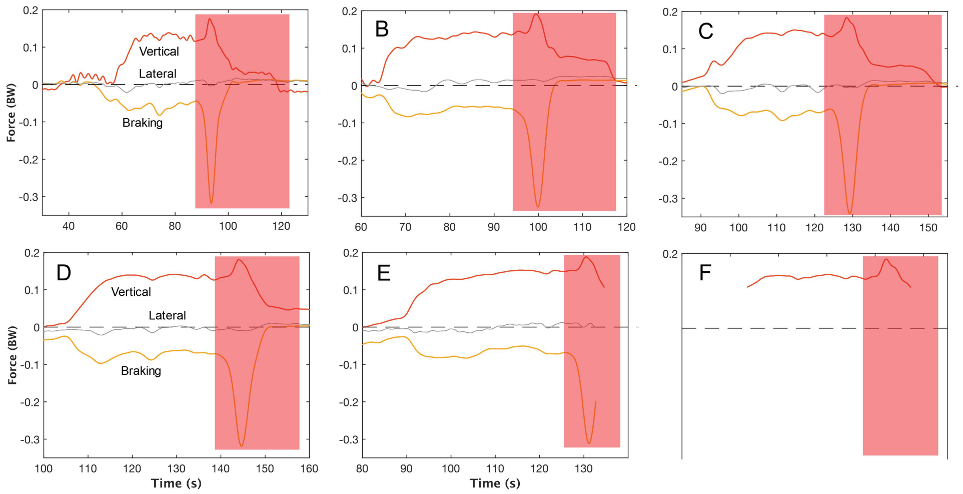

Figure S2. All trials obtained from the inert nylon rope dragged across the force-sensing dowel. The red region shows when the rope fell off the dowel adjacent to the force-sensing dowel, generating substantial forces due to inertial motion. This region was excluded from our analysis.

Journal of Experimental Biology • Supplementary information

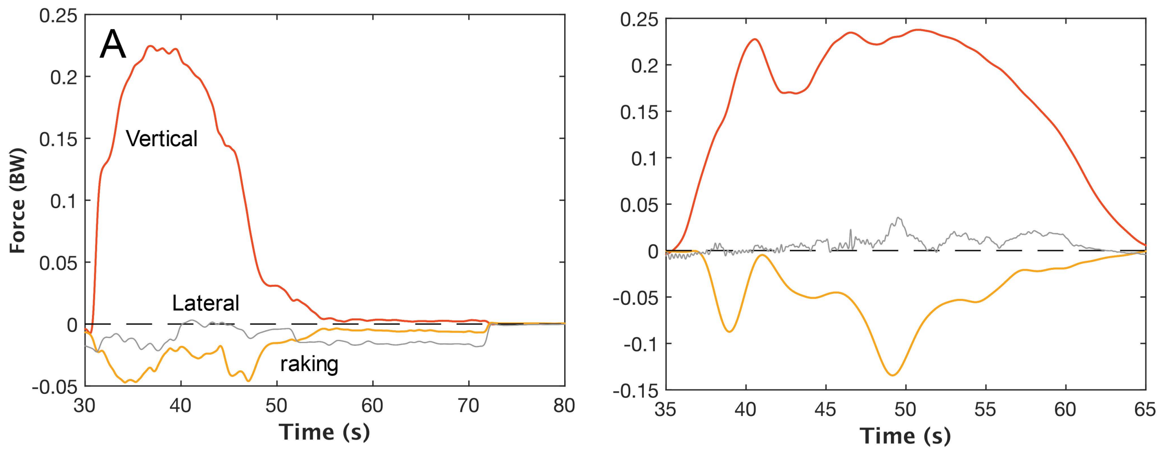

Fig. S3. All trials obtained that have purely braking force (or nearly so). Trials are from snake 4 (A) and snake 3 (B).

Journal of Experimental Biology • Supplementary information

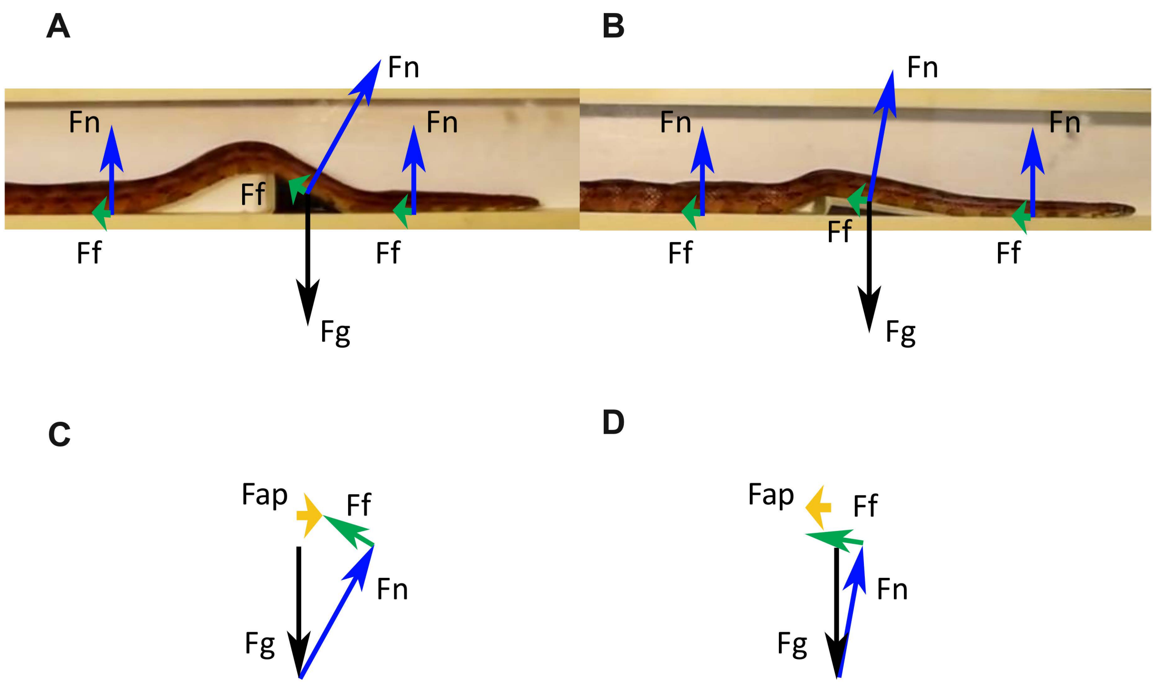

Fig. S4. A) Reaction forces for the corn snake vertically undulating against the 33 wedge at 30º. Fap-anteroposterior force, Ff-frictional force, Fg-gravitational force, Fn-normal 34 force. B) Reaction forces for the corn snake using concertina against the wedge at 10º. C) Body 35 diagram summarizing the forces from A showing a net propulsive force. D) Body diagram 36 summarizing the forces from B showing a net braking force.

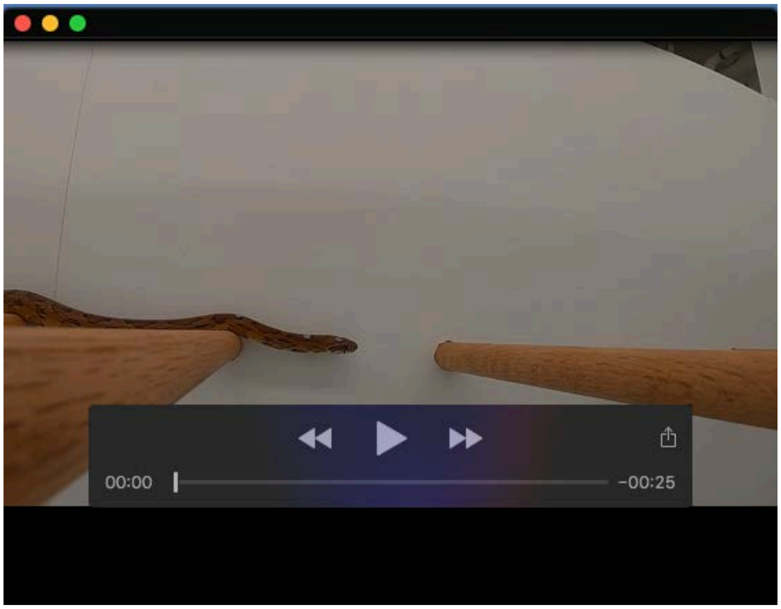

Movie 1. Corn snake performing vertical undulations in lateral view across the force-sensing dowel.

Journal of Experimental Biology • Supplementary information

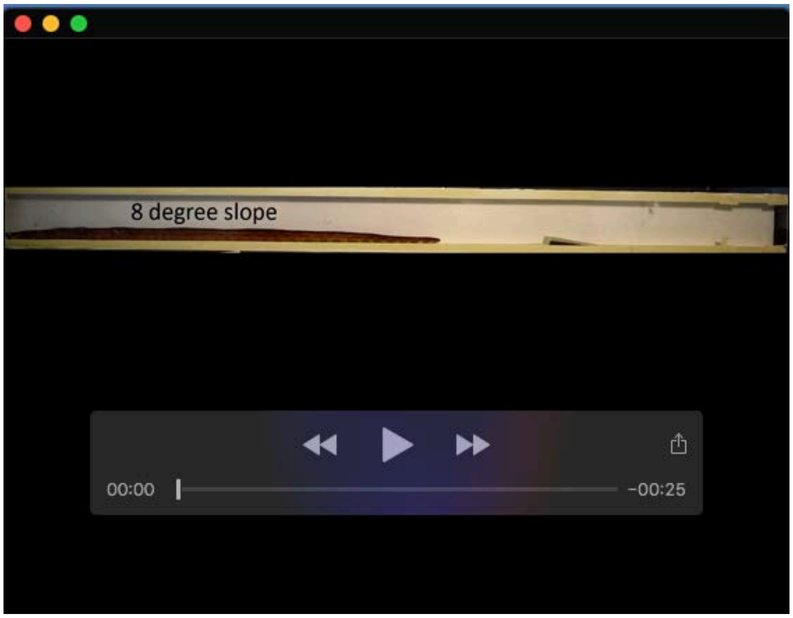

Movie 2. Corn snake in lateral view within a tunnel made of PVC boards showing two trials. In the first trial both prior to and while crossing the wedge (sloped at 8°) the corn snake performs concertina locomotion. Following this trial, prior to the wedge (sloped at 30°), the corn snake performs concertina locomotion and transitions to vertical undulations after encountering the wedge.

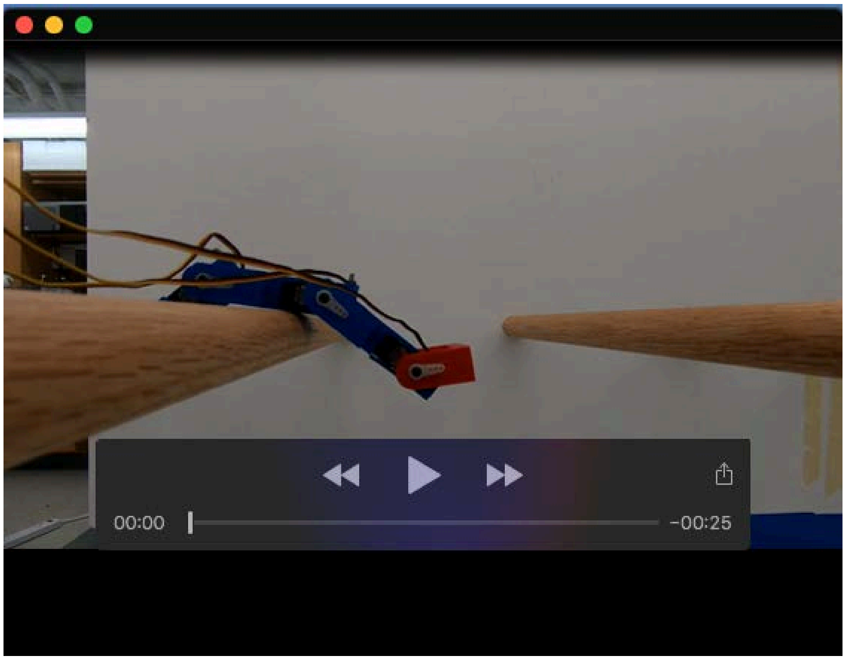

Movie 3. Snake robot performing vertical undulation in lateral view.

Supplementary Materials and Methods 1. Code used to control the snake robot in the software Python.

```
#Code for the software Python used to control the snake robot.

import serial

import sys

import time

import math

import numpy

import scipy



#variables

x=scipy.arange(0, 35, 1) #time interval for robot, 35 seconds total

a=600 #amplitude

#t=x #'time'

d=0 #phase shift

z=1450 #translation up/down to ‘zero’ servo motors

z1=1500

z2=1475

z3=1425



#Motor0

d0=17.5

#Motor1

d=16
```

Journal of Experimental Biology • Supplementary information

```
#Motor2

d1=14.5

#Motor3

d2=13

#Motor4

d3=11.5

#Motor5

d4=10

#Motor6

d5=8.5

#Motor7

d6=7

#Motor8

d7=5.5

#Motor9

d8=4

#Motor10

d9=2.5

#Motor11

d10=1


#equations separated by motor

y=((a*(numpy.sin(x+d0)))+z1)
```

```
y1=((a*(numpy.sin(x+d)))+z3)

y2=((a*(numpy.sin(x+d1)))+z2)

y3=((a*(numpy.sin(x+d2)))+z)

y4=((a*(numpy.sin(x+d3)))+z)

y5=((a*(numpy.sin(x+d4)))+z)

y6=((a*(numpy.sin(x+d5)))+z1)

y7=((a*(numpy.sin(x+d6)))+z1)

y8=((a*(numpy.sin(x+d7)))+z1)

y9=((a*(numpy.sin(x+d8)))+z1)

y10=((a*(numpy.sin(x+d9)))+z1)

y11=((a*(numpy.sin(x+d10)))+z)



#to read lynxmotion

ssc32 = serial.Serial('/dev/cu.usbserial-A10731HN', 9600, timeout=1.0)


#servo motors information

sin=str(y)

#motor 0

motor='#0 P'

extra='T600 \r'


#motor 1
```

Journal of Experimental Biology • Supplementary information

```
motor1='#1 P'

#motor 2

motor2='#2 P'

#motor 3

motor3='#3 P'

#motor 4

motor4='#4 P'

#motor 5

motor5='#5 P'

#motor 6

motor6='#6 P'

#motor 7

motor7='#7 P'

#motor 8

motor8='#8 P'

#motor 9

motor9='#9 P'

#motor 10

motor10='#10 P'

#motor 11

motor11='#11 P'


print("start")
```

Journal of Experimental Biology • Supplementary information

```
#delay before running, and motor delay prior to next position
time.sleep(2)

r = y.shape
for robot in range(0,r[0]):
    #motor0
    final=motor+str(y[robot])+' '+extra
    ssc32.write(bytes(final, 'ASCII'))
    time.sleep(.05)

    #motor1
    final=motor1+str(y1[robot])+' '+extra
    ssc32.write(bytes(final, 'ASCII'))
    time.sleep(.05)

    #motor2
    final=motor2+str(y2[robot])+' '+extra
    ssc32.write(bytes(final, 'ASCII'))
    time.sleep(.05)

    #motor3
    final=motor3+str(y3[robot])+' '+extra
```

Journal of Experimental Biology • Supplementary information

```
ssc32.write(bytes(final, 'ASCII'))
time.sleep(.05)

#motor4
final=motor4+str(y4[robot])+' '+extra
ssc32.write(bytes(final, 'ASCII'))
time.sleep(.05)

#motor5
final=motor5+str(y5[robot])+' '+extra
ssc32.write(bytes(final, 'ASCII'))
time.sleep(.05)

#motor6
final=motor6+str(y6[robot])+' '+extra
ssc32.write(bytes(final, 'ASCII'))
time.sleep(.05)

#motor7
final=motor7+str(y7[robot])+' '+extra
ssc32.write(bytes(final, 'ASCII'))
time.sleep(.05)
```

Journal of Experimental Biology • Supplementary information

```
    #motor8
    final=motor8+str(y8[robot])+' '+extra
    ssc32.write(bytes(final, 'ASCII'))
    time.sleep(.05)

    #motor9
    final=motor9+str(y9[robot])+' '+extra
    ssc32.write(bytes(final, 'ASCII'))
    time.sleep(.05)

    #motor10
    final=motor10+str(y10[robot])+' '+extra
    ssc32.write(bytes(final, 'ASCII'))
    time.sleep(.05)

    #motor11
    final=motor11+str(y11[robot])+' '+extra
    ssc32.write(bytes(final, 'ASCII'))
    time.sleep(.05)


time.sleep(2)
```

Journal of Experimental Biology • Supplementary information

```
#return robot to flat position

ssc32.write(bytes('#0 P1500 T500 #1 P1425 T500 #2 P1475 T500 #3 P1450 T500 #4 P1450
T500 #5 P1450 T500 #6 P1500 T500 #7 P1500 T500 #8 P1500 T500 #9 P1500 T500 #10 P1500
T500 #11 P1450 T500 \r', 'ASCII'))


print("end")

ssc32.close()
```

Journal of Experimental Biology • Supplementary information
